# Familial Focal Segmental Glomerulosclerosis With Late-Onset Presentation and R229Q/R291W Podocin Mutations

**DOI:** 10.3389/fgene.2020.533373

**Published:** 2020-09-16

**Authors:** Michelle T. P. Riguetti, Patrícia Varela, Danilo E. Fernandes, M. Goretti Polito, Fernanda M. Casimiro, João B. Pesquero, Gianna Mastroianni-Kirsztajn

**Affiliations:** ^1^Department of Medicine, Division of Nephrology, Federal University of São Paulo, São Paulo, Brazil; ^2^Center for Research and Molecular Diagnostic of Genetic Diseases – Department of Biophysics, Federal University of São Paulo, São Paulo, Brazil

**Keywords:** FSGS, gene polymorphism, glomerulonephritis, podocin, *NPHS2*

## Abstract

**Introduction:**

Pathogenic variants in different genes have been described as involved in the development of familial focal segmental glomerulosclerosis (FSGS). A more precise genotype–phenotype correlation would be helpful to better characterize the clinical and laboratorial manifestations of this disease, as well as response to treatment. We analyzed podocin (*NPHS2*) gene variants in 50 members of four generations of a family with late-onset presentation of glomerular disease.

**Results and Discussion:**

The *NPHS2* gene variants R229Q and/or R291W were detected in several individuals, and the phenotype of FSGS with progressive loss of renal function was observed in all the family members carrying both mutations simultaneously. Patients manifested ongoing proteinuria over the years and progressive loss of renal function, which in three women culminated in renal replacement therapy by the 4th decade of life. In two affected patients with nephrotic syndrome, remission was not reached by the use of corticosteroids and other immunosuppressive drugs. The R229Q variant was pathogenic only when trans-associated with specific mutations, as the R291W variant in this family.

**Conclusion:**

Coexistence of the two *NPHS2* variants R229Q and R291W in compound heterozygosis was a determinant of the FSGS phenotype. The presence of these variants alone in heterozygosis did not cause significant proteinuria.

## Introduction

Focal segmental glomerulosclerosis (FSGS) is a major cause of end-stage renal disease (ESRD) and deserves particular attention as a progressive chronic kidney disease (CKD) that affects predominantly children, adolescents, and young adults. This disease is the most frequent glomerulopathy in Brazil when registries of renal biopsy are evaluated (Polito et al., 2010). FSGS corresponds to a spectrum of glomerular diseases with a common histological pattern caused by a variety of insults, as infectious agents, circulating permeability factors (Fogo, 2015) and genetic determinants; some of them already identified.

In fact, several pathogenic variants described in more than 50 genes are involved in the development of part of the cases of FSGS and were identified in familial as well as in sporadic cases (Monteiro et al., 2006).

Although familial presentation of FSGS is known for decades, detailed clinical data including presentation of the disease, course, response to treatment, and prognosis are still scarce, as well as adequate medical indication for requesting mutation tests in cases of FSGS.

Considering that the detailed study of families with inherited FSGS can contribute to understanding of those forms of FSGS with genetic origin, we present a four-generation family with 50 studied members that were screened for renal dysfunction and *NPHS2* mutations.

## Materials and Methods

### Ethical Approval and Informed Consent

All procedures performed in studies involving human participants were in accordance with the ethical standards of the institutional and/or national research committee at which the studies were conducted (CEP/UNIFESP approval number 0602/09) and with the 1964 Helsinki declaration and its later amendments or comparable ethical standards.

Informed consent was obtained from all individual participants included in the study.

### Patients

We analyzed 50 members of a four-generation family assisted by the Glomerulopathies Section of the Division of Nephrology of UNIFESP, with two members with biopsy-proven FSGS.

Peripheral blood samples were collected using EDTA as an anticoagulant for gene sequencing, except in the case of the young children, when swab collection was used. Serum and urine were used for renal evaluation tests. In adults, serum creatinine, estimated glomerular filtration rate (CKD-EPI equation), urinary albumin/creatinine and/or protein/creatinine ratios, and/or 24-h proteinuria were determined, and in the young children only the described urinary tests were performed.

## Methods

### Isolation of Genomic DNA and PCR Analysis

Genomic DNA was extracted from peripheral blood sample collected in EDTA using DNAzol^®^ kit (Invitrogen). Fragments containing all the exons and the flanking regions from *NPHS2* were amplified with specific primers (Exxtend, Paulinia) by PCR, and 10 μL of each sample was subjected to electrophoresis on 1% agarose gel. Amplification products were identified according to the expected molecular weight.

The eight exons of *NPHS2* gene were amplified by PCR for the index patient and the exons 5 and 7 for the other members of the family.

### Sequencing of DNA and Bioinformatic Analysis

Each amplicon was purified using ExoSap^®^ IT (GE Healthcare) according to the manufacturer’s instructions and sequenced using the BigDye Terminator v3.1 cycle sequencing kit and ABI Prism 3500xl Genetic Analyzer sequencer. Sequences were compared with the reference sequence NG_007535^1^ and confirmed by reverse strand sequencing. Variants were reviewed and annotated using dbSNP (single-nucleotide polymorphism database^2^ and HGMD (Human Genome Mutation Database professional, biobaseinternational.com/product/hgmd).

### Renal Function Markers

Creatinine (in urine and serum) was determined by alkaline picrate method, proteinuria (random and 24-h proteinuria) by immunoenzymatic assay method, and albuminuria by immunoturbidimetry. Albumin/creatinine (ACR) and protein/creatinine ratios (PCR) were calculated, and glomerular filtration rate based on serum creatinine levels was estimated by the Chronic Kidney Disease Epidemiology Study (CKD-EPI) formula.

## Results

**FIGURE 1:**
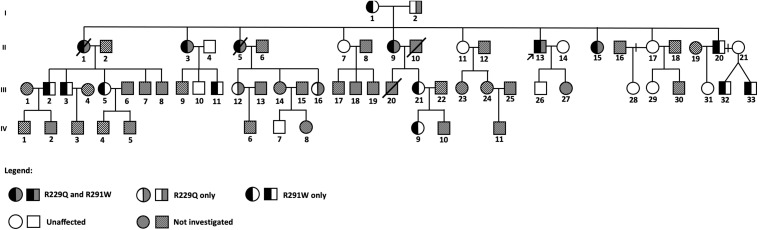
Pedigree of a large family with familial FSGS.

Biological material was collected from members of a family in which six siblings out of 10 (representing the 2nd generation) had CKD (Figure 1). The laboratory analysis to assess renal function was performed in 48 subjects and sequencing of *NPHS2* in 30 out of 50 individuals. The index case II-13 (shown by an arrow) was admitted in our service when he was 19 years old presenting nephrotic range proteinuria. His kidney biopsy corresponded to FSGS (segmental hyalinosis and sclerosis in 6, and global sclerosis in 2 out of 12 glomeruli, as well as focal tubular atrophy and interstitial fibrosis). Renoprotective measures (angiotensin-converting-enzyme inhibitors and angiotensin II receptor blockers, statins, and other medications as necessary) were maintained in the patient II-13 along 23 years of follow-up; steroid therapy was initiated when he presented nephrotic syndrome, and other immunosuppressive medications were administered in different periods due to nephrotic syndrome. By admission, 24-h proteinuria was 3.52 g/24 h and during the follow-up ranged from 1.48 to 8.86 g, and the lowest levels were observed during the use of calcineurin inhibitor. Estimated GFR was normal by the onset of disease and at the last follow-up was 54 mL/min/1.73 m^2^.

The index case II-13 was submitted to genetic analysis that included the following four genes known to cause FSGS: *NPHS2*, *ACTN4*, *TRPC6*, and *INF-2*. He presented two variants in *NPHS2* (R229Q and R291W), and only these mutations were analyzed in the other family members.

His parents were not consanguineous, and both of them did not present renal disease. His mother (I-1) has diabetes and hypertension and carried only the variant R291W in heterozygosis. His father (I-2) had hypertension and died before the beginning of this study, but we found no evidence of proteinuric renal disease or end-stage renal disease (ESRD) based on information provided by the family members.

The index patient and his nine siblings constituted the 2nd generation of this family, in which three individuals had proteinuric glomerular disease, identified as FSGS by renal biopsy performed in the index patient (II-13) and one sister (II-9). Four other sisters (II-1, II-3, II-5, and II-15) presented ESRD, three of them (II-1, II-3, and II-5) were submitted to renal transplantation (without developing recurrent FSGS in their renal grafts). Then, genetic analysis for mutations in the *NPHS2* gene was performed, and all the four living family members with renal disease (II-3, II-9, II-13, and II-15) presented both R229Q and R291W variants in the *NPHS2* gene.

In two patients of the 2nd generation (II-9 and II-13) with nephrotic syndrome, remission was not reached by the use of corticosteroids and other immunosuppressants, but proteinuria decreased while using calcineurin inhibitor cyclosporine.

The 3rd generation is composed of 33 individuals, and the age of the individuals by the time of data collection varied from 4 to 36 years. Sanger sequencing of the *NPHS2* gene was performed in 14 of them; the variant R229Q was present in two (III-12 and III-16) and R291W in seven members (III-2, III-3, III-5, III-11, III-21, III-32, and III-33) of this generation. Two females (III-12 and III-16) presenting the variant R229Q in heterozygosis (26- and 33-year-old daughters of a patient with FSGS) have no evidence of renal disease until that moment. However, two members (III-11 and III-16) had microalbuminuria (levels above 30 mg/g).

In the 4th generation, subjects were 2–16 years old at the moment of sample collection, and 1 out of 11 relatives had the variant R291W: an 11-year-old member (IV-9), daughter of a healthy female member of this family. This girl presents persistent glomerular hematuria and microalbuminuria and is under close follow-up.

Clinical and laboratory data are shown in Table 1.

**TABLE 1:** Clinical and laboratorial characteristics of the family members with identified *NPHS2* variants.

Generation	Subject	Age (years)	Gender	Variant	Serum creatinine (mg/dL)	Serum albumin (g/dL)	Urinary albumin/creatinine ratio (mg/g)	Urinary protein/creatinine ratio (g/g)
I	1	74	Female	R291W	1.2	5.2	8.0	0.0
II	1*	57	Female	R291W + R229Q	NA	NA	NA	NA
II	3**	55	Female	R291W + R229Q	1.7**	4.5	<5.00	0.0
II	5*	53	Female	R291W + R229Q	NA	NA	NA	NA
II	9	49	Female	R291W + R229Q	0.8	2.8	2359.1	2.99
II	13	40	Male	R291W + R229Q	2.5	2.9	2109.1	1.99
II	15	38	Female	R291W + R229Q	0.4	3.6	3353.2	3.81
II	20	33	Male	R291W	NA	NA	NA	NA
III	2	36	Male	R291W	1.0	5.2	8.0	0.0
III	3	31	Male	R291W	0.9	4.8	<5.00	0.0
III	5	30	Female	R291W	0.8	4.4	20.6	0.0
III	11	19	Male	R291W	1.0	5.3	46.5	0.07
III	12	33	Female	R229Q	0.8	4.5	<5.00	0.0
III	16	26	Female	R229Q	0.9	5.1	37.4	0.09
III	21	30	Female	R291W	0.9	4.9	18.3	0.0
III	32	12	Male	R291W	NA	NA	<5.00	0.0
III	33	12	Male	R291W	NA	NA	16.8	0.0
IV	9	11	Female	R291W	0.5	5.0	147.6	0.19

Abbreviations: ESRD, end-stage renal disease; NA, not available; Tx, renal transplantation. *ESRD + Tx; died after kidney transplantation. **ESRD + Tx; renal function after kidney transplantation.

In summary, the phenotypes of FSGS or proteinuric glomerular disease were observed in each member that carried both variants. The diagnosis was done at 19 years of age or later, manifested as ongoing proteinuria over the years and progressive loss of renal function, which ended in renal replacement therapy (RRT) in three women after about 20 years of renal disease diagnosis. When only one of the mutations was present, manifestation of glomerular disease was not observed, except in a diabetic young man of the 3rd generation (III-11) and an 11-year-old girl of the 4th generation (IV-9).

## Discussion

The combined mutation R229Q/R291W in compound heterozygosity was previously described by other groups (Zhang et al., 2004; Tory et al., 2014; Stráner et al., 2018). However, in the present study we highlight the clinical and laboratory presentation of the FSGS correlated phenotype in a long-term follow-up of several family members carrying both variants in compound heterozygosity, as well as the clinical picture of those members carrying only one of the mutations.

Many gene variants have been associated with FSGS and steroid-resistant nephrotic syndrome (SRNS) for the last decades, but the applicability of genetic testing in clinical practice is still uncertain, despite the reports on genetically caused glomerular diseases (Monteiro et al., 2006). According to recent studies, FSGS can be caused by more than 50 gene mutations, with a monogenic inheritance pattern (Preston et al., 2019).

*NPHS2* is the most frequently mutated of these genes causing FSGS, and it is also a determinant of monogenic forms of SRNS. The *NPHS2* gene has eight exons that encode for podocin, a membrane protein that is located exclusively in the slit diaphragm (Tory et al., 2014). Podocin accumulates in dimeric or oligomeric forms in lipid raft microdomains at the podocyte slit diaphragm, which is an essential component of the glomerular filtration barrier. Many reported mutations in this gene encode truncated proteins, suggesting that FSGS results from a loss of function of *NPHS2* (Pollak, 2002).

In the family here evaluated, *NPHS2* gene mutations were detected in the 2nd-generation members and those with both variants R229Q/R291W together presented renal disease that was diagnosed in 7 out of 10 siblings, generally in adulthood. They had glomerular disease with progressive loss of renal function. Significant proteinuria appeared in the affected 2nd-generation family members when they were 19 years old or later. The remaining three siblings did not present renal involvement, as well as no mutations in *NPHS2*.

Mikó et al. (2018) described the variant p.R229Q as the most commonly found *NPHS2* mutation in the general population and as the first human variant whose pathogenicity is dependent of another associated mutation. It means that p.R229Q causes FSGS if it is trans-associated with specific mutations that affect the protein region spanning residues 270–351. In fact, Feltran et al. (2017) showed that the combination of the R229Q variant with other mutations located in residues 260 and 310 led three children to ESRD. Here, we present data that endorses the progressive nature of the glomerular disease, but with a later onset.

It is known that podocin oligomerization occurs exclusively through the C-terminal tail and influences the localization of podocin in a complex form. Stráner et al. (2018) reported that R229Q makes the C-terminal helical tail less flexible, which makes it prone to form abnormal heterodimers with some podocin mutants such as R291W, affecting its dimerization, thus explaining the dominant negative effect of these variants.

According to Tory et al. (2014) and Stráner et al. (2018), the association between R229Q and R291W exerts a dominant negative effect on p.R229Q podocin by blocking its membrane trafficking through an altered heterooligomerization. Then, the R229Q podocin is retained in cytoplasmic compartments, leading to a significant loss of podocin function with a more severe phenotype of renal disease and progression to CKD5, as observed in the family we described, since three individuals required renal transplantation and none of the others responded to immunosuppressive treatment. Nishibori et al. (2004) have demonstrated that some missense mutations in the *NPHS2* gene, as the R291W, not only lead to misfolding and mislocalizating the mutated podocin, but they can also interfere with slit diaphragm structure and function by altering the proper trafficking of nephrin to the plasma membrane.

Zhang et al. (2004) evaluated the *in vivo* expression of podocyte slit diaphragm-associated proteins in nephrotic patients with NPHS2 mutations and described patients with compound heterozygous mutations in the *NPHS2* gene, among them R291W/R229Q. They concluded that an abnormal podocin changes the distribution of nephrin, CD2AP, and alpha-actinin.

The same variants R291W and R229Q were found in individuals of the 3rd and 4th generation of this family, but not together. Thus, our study corroborates the pathogenicity of the inheritance of both R229Q and R291W variants together in compound heterozygosity. It was observed that such variants alone in heterozygosity did not cause glomerular disease in individuals of the 3rd and 4th generations and some of them presented only non-persistent microalbuminuria, except for a girl of the 4th generation with the R291W variant that had persistent microalbuminuria and hematuria. Therefore, based on these findings, we can suggest that in this family neither R291W nor R229Q variants alone in heterozygosity could be definitely related to microalbuminuria. Although a previous large study has reported an association between R229Q variant and development of microalbuminuria in the general population (Pereira et al., 2004), others did not confirm such finding (Köttgen et al., 2008).

All family members are invited to perform a checkup in our service at regular intervals. As treatment in this family aims renoprotection, all members with proteinuria are submitted to blockade of the renin angiotensin system, control of blood pressure, and hyperlipidemia, as well as of all metabolic disorders eventually diagnosed. In addition, the first members to present nephrotic syndrome were submitted tentatively to steroid therapy and to other immunosuppressive treatments. A better control of proteinuria levels during calcineurin inhibitor therapy was observed for some months, as previously described by others (Klaassen et al., 2015) in patients carrying *NPHS2* mutations treated with cyclosporine, but not with steroid, cyclophosphamide, or sodium mycophenolate.

Renal transplantation is a matter of concern in patients with FSGS as the general rate of recurrence is high (Uffing et al., 2018). Although three women of the 2nd generation were submitted to renal transplantation, none of them presented recurrent FSGS. In fact, recurrence is rare in familial FSGS, but it has also been described (Pollak, 2014).

Establishing a distinction between pathogenic and benign associations with R229Q is important, but difficult in clinical practice because the phenotype of NPHS2-related SRNS/FSGS is observed also in patients without NPHS2 mutations (Sadowski et al., 2015) and the p.R229Q variant is common in the European, South Asian, African, and Latino-American population (Mikó et al., 2018). It is of note that the population frequency of R229Q is 0.02 (Brazilian Online Archive of Mutations – Abraom) and 0.03 (Gnomad/Exac), while the frequency of R291W is not described in Brazil and is 0.00002 (Gnomad/Exac) worldwide, respectively.

Genetic forms of FSGS may follow different patterns of inheritance that can be difficult to identify, particularly in small families (Tonna et al., 2008). Nevertheless, the study of well-evaluated families with FSGS can contribute to understanding the genetic forms of such glomerular disease. In addition, the large number of family members as well as the long follow-up time allowed us to better characterize this family.

Considering cost–benefit, especially in low-income countries, and some authors reports (Monteiro et al., 2006; He et al., 2007), genetic investigation has still limited clinical applicability in adults with sporadic FSGS. On the other hand, it is certainly useful in case of early onset FSGS or clear history of familial glomerular disease, like the family here discussed, in which genetic analysis is rather important, contributing at least for the treatment choice and genetic counseling, including embryo selection according to the type of inheritance.

## Data Availability Statement

The datasets for this article are not publicly available due to concerns regarding participant/patient anonymity. Requests to access the datasets should be directed to the corresponding author.

## Ethics Statement

Written informed consent was obtained from the individuals and minors’ legal guardian/next of kin for the publication of any potentially identifiable images or data included in this article.

## Author Contributions

MTPR, PV, DEF, MGP, and FMC collected data and performed the analyses under the supervision of JBP and GM-K. All the authors had access to the data and worked on the final version of this manuscript.

## Conflict of Interest

The authors declare that the research was conducted in the absence of any commercial or financial relationships that could be construed as a potential conflict of interest.

## Footnotes

1 http://www.ncbi.nih.gov

2 http://ncbi.nlm.nih.gov/projects/SNP/
